# Microwave ablation for diffuse adenomyosis leading to multiple complications after hysterectomy: A case report and literature review

**DOI:** 10.1097/MD.0000000000037701

**Published:** 2024-04-05

**Authors:** Xiuchun Yang, Wenhui Zhao, Shujuan Chen, Jinhong Yang

**Affiliations:** a School of Nursing, Weifang Medical University, Weifang, People’s Republic of China; b Gynecology Ward, Yidu Central Hospital, Weifang, People’s Republic of China; c Department of Oncology, Weifang People’s Hospital, Weifang, People’s Republic of China.

**Keywords:** ablation, adenomyosis, complication, HIFU, hysterectomy

## Abstract

**Rationale:**

Hysterectomy after microwave ablation (MWA) is more difficult than conventional surgery which increases the probability of postoperative complications due to MWA’s collateral thermal damage to nearby intestines. Here we report a case of multiple postoperative complications after hysterectomy following MWA.

**Patient concerns:**

A 44-year-old female was admitted due to progressive abdominal pain during menstruation for 30 years and no relief 1 year after MWA. Hysterectomy was performed. Intraoperative findings: pelvic inflammatory exudation; the uterus and the left adnexa were extensively and densely adhered to the intestine, bladder, pelvic wall and surrounding tissues; the local tissue of the uterus was brittle and dark yellow. Intestinal obstruction, abdominal infection and urinary fistula occurred after hysterectomy.

**Diagnoses:**

1. Adenomyosis. 2. Endometrial polyps. 3. Left chocolate cyst of ovary. 4. Pelvic adhesions. 5. Pelvic inflammation.

**Interventions:**

The patient underwent intestinal obstruction catheter implantation, ultrasound-guided pelvic fluid mass puncture drainage, right kidney puncture and fistula drainage, right ureteral bladder replantation, and right ureteral stent implantation.

**Outcomes:**

After 48 days of comprehensive treatment, the patient was cured and discharged.

**Lessons:**

Microwave ablation has a poor therapeutic effect on diffuse adenomyosis, and should avoid excessive ablation during the ablation process.

## 1. Introduction

Adenomyosis is a benign disease caused by the invasion of the endometrial glands or stroma into the myometrium.^[1]^ It is a common gynecological disorder in women of childbearing age, with an average incidence of 20% to 30%.^[2]^ The main clinical symptoms are menorrhagia, dysmenorrhea, uterine enlargement and infertility, and it can also lead to anemia, which seriously affects the women’s quality of daily life and mental health.^[3]^ Hysterectomy remains the most popular and radical treatment for adenomyosis.^[4]^ However, hysterectomy is not suitable for women with demands of fertility.^[5]^ In recent years, ultrasound-guided microwave ablation (MWA) has provided a minimally invasive method to preserve the uterus, especially for women who have reproductive demands. It was reported MWA for adenomyosis could relieve the symptoms of adenomyosis and not affect ovary function.^[6,7]^ However, MWA is a thermal ablation method that has the potential risk of collateral thermal damage to nearby intestines.^[8]^ Especially for women with diffuse adenomyosis, excessive ablation and endometrial destruction may lead to postoperative pelvic infection or uterine adhesion.^[9]^ Besides, MWA method is technically challenging because of surrounding organs interference.

Here, we present a patient who had no relief of symptoms after MWA, and underwent hysterectomy. However, due to the dense adhesion between the uterus and surrounding tissues following by MWA thermal damage, the patient was complicated by postoperative intestinal obstruction, abdominal infection and urinary fistula.

## 2. Case presentation

**Figure 1.**
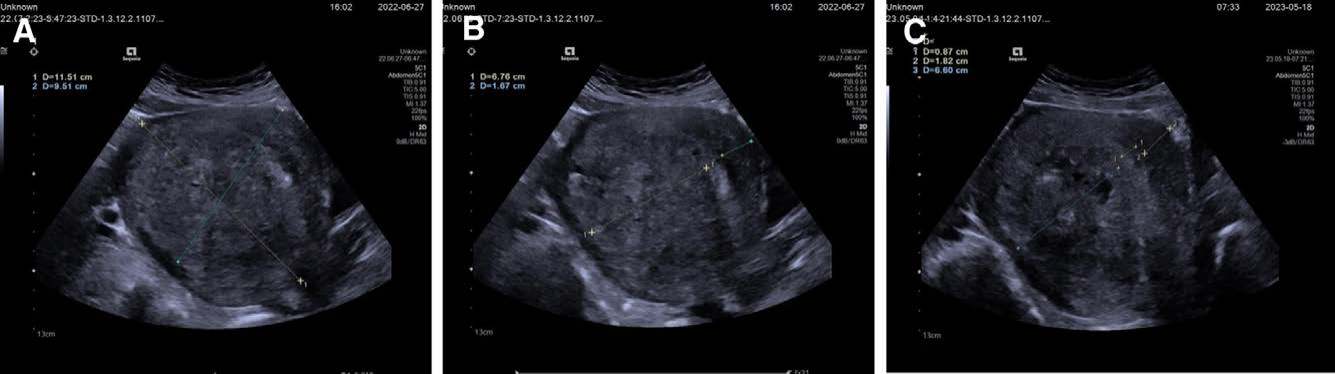
(A–C) Gynecological ultrasound showed uterus size 11.51 × 9.51 cm (A). Pre-MWA uterine wall thickness: anterior wall 1.67 cm, posterior wall 6.67 cm (B). Post-MWA 11 months: anterior wall 1.82 cm, posterior wall 6.60 cm (C).

A 44-year-old female was referred to our hospital for progressive lower abdominal pain during menstrual period for 30 years. She had a history of microwave ablation due to adenomyosis 1 year ago (Fig. 1A and B), but there was no significant relief in lower abdominal pain after MWA and the menstrual volume remained unchanged compared to before. B ultrasound showed uterine enlargement, adenomyosis (mainly posterior wall), heterogeneous echogenic area of the posterior wall of the uterus (Fig. 1C). Gynecological examination showed cervical hypertrophy, uterus anterior position, like 5+ months pregnancy with hard texture. Hysteroscopy examination showed multiple polypoid protrusions in the uterine, with no abnormal blood vessels on the surface, with endometrial thickness and no visible bilateral fallopian tube openings. Pathological examination result showed that benign lesions were considered. Preoperative diagnosis were adenomyosis of the uterus and endometrial polyps.

**Figure 2.**
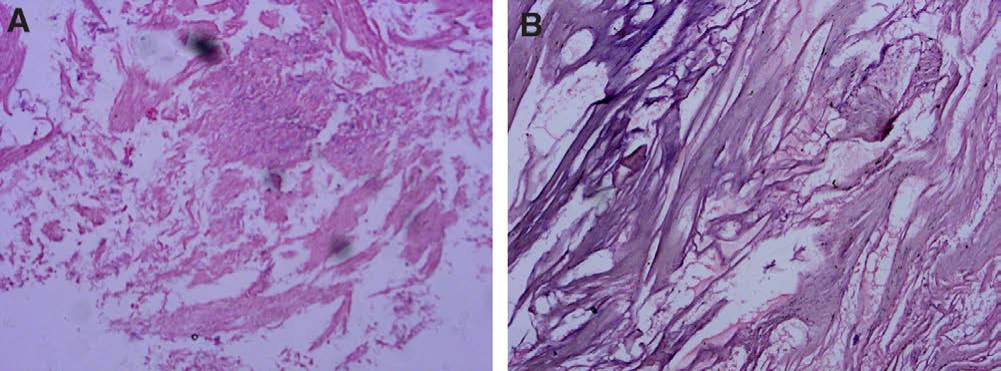
(A and B) Pathology shows partial smooth muscle necrosis.

Laparoscopic total hysterectomy with double salpingectomy was performed due to the patient’s indication for surgery: severe symptoms, no fertility requirements, and refusal of alternative options. Intraoperative exploration revealed inflammatory exudation in the pelvic cavity, irregular shape of the uterine with size about 5+ months of pregnancy, and the left ovarian tumor approximately 6 × 6 × 5 cm, and the uterus, left adnexa, intestinal tract, bladder, pelvic wall, and surrounding tissues were widely and densely adhered. Laparoscopic total hysterectomy and double salpingectomy, as well as left ovarian tumor removal were performed combined with pelvic adhesions lysis. After removing the uterus, it was found that the local tissue of the uterus was crispy and dark yellow in color; pathology shows partial smooth muscle necrosis (Fig. 2A and B). The entire surgical process was difficult but smooth, with approximately 50 mL of intraoperative bleeding. Due to the presence of inflammatory exudation in the patient’s pelvic cavity, therapeutic antibiotics such as levofloxacin were administered postoperatively, followed by fluid replacement, electrocardiogram monitoring, and continuous abdominal drainage.

**Figure 3.**
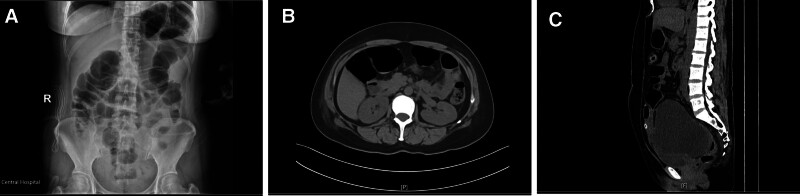
(A–C) Abdominal X-ray: Intestinal gas accumulation with atypical small fluid (A). Abdominal CT: intestinal dilation and gas accumulation (B). Abdominal and pelvic CT: pelvic encapsulated fluid accumulation (C).

However, abdominal distension occurred on the first day after surgery, and intestinal obstruction was diagnosed on the third day after surgery (Fig. 3A and B). On the 15th day after surgery, a CT scan of the entire abdomen and pelvic cavity was performed, which showed fluid accumulation in the abdomen and pelvic cavity, and a small amount of fluid accumulation in the chest cavity, and pelvic encapsulated effusion (Fig. 3C). On the 21st day after surgery, the patient developed pale yellow vaginal discharge and was diagnosed right lower ureteral fistula and vaginal fistula. After undergoing right ureteral bladder replantation and right ureteral stent placement under general anesthesia, symptomatic treatments such as continuous catheterization and infection prevention were given postoperatively. On the 44th day after hysterectomy, the patient’s condition was stable without vaginal bleeding or other discomfort. At the telephone follow-up 2 months later, the patient reported that everything was in good condition with no recurrence of abdominal pain and quality of life was improved.

## 3. Discussion

To the best of our knowledge, this is the first report describing a case where a total hysterectomy was performed after poor postoperative results of microwave ablation for diffuse adenomyosis, but a series of complications occurred due to adhesions and tissue damage followed by MWA.

There are 2 main types of adenomyosis: diffuse and focal. Adenomyosis is classified as diffuse when the endometrial glands or stroma are dispersed within the myometrium and as focal when the lesions are localized.^[4]^ In this case, the anterior wall of uterus was 1.67cm thick and the posterior wall of uterus was 6.76cm thick. It diffuse adenomyosis of uterus (mainly posterior wall). The principle of MWA is to use microwave radiators to convert electromagnetic wave energy into radiant energy, resulting in an instant increase in tissue temperature, degeneration, solidification and necrosis, so as to achieve the purpose of treating the disease without removing the uterus.^[10]^ Since June 2006, the ablation technique of adenomyosis has been applied in many centers in China. A number of studies have confirmed that MWA has a significant effect on focal uterine adenomyosis, reducing the size of the uterus and improving the symptoms of dysmenorrhea and menorrhagia in patients.^[6–8]^ However, it was also reported that the effect of MWA was not good and the incidence of complications was high in the treatment of diffuse adenomyosis.^[11,12]^ The case’s symptoms did not relieve and complications occurred after MWA. The reason may be that it is difficult to focus on obvious lesion because of diffuse thickening of the uterus. Ablation could not effectively deal with the hidden lesion, the lesion close to the intima layer or the serosa layer, which would easily lead to incomplete ablation of the lesion.^[12]^ At the same time, excessive ablation in pursuit of ablation rate may easily lead to damage of surrounding tissues. The key to ablation is to achieve sufficient ablation while avoiding damage to the surrounding tissue.^[13]^ Before operation, the application of MWA should be carefully evaluated according to the characteristics of the cases, and the individualized ablation method should be selected.

Age should be another aspect that should be carefully evaluated before MWA operation. In this case, the patient is older. It is recommended to select appropriate power parameters according to different ages in terms of heat use. The efficacy of MWA in the treatment of adenomyosis is related to a variety of factors, of which, age and ablation rate are related factors.^[14]^ Older patients have lower estrogen levels than younger patients, and lesion of the same size requires less energy. Especially in perimenopausal patients, lesions are more likely to ablate. Large breakthroughs are more likely to occur during ablation.

In addition, the higher the ablation rate and the wider the ablation range, the more likely the damage of the serosal layer and surrounding organs will occur. In this case, the postoperative complications were visible during the surgery. For example, (1) the uterus and the left adnexa were extensively and densely adhered to the bowel, bladder, pelvic wall and surrounding tissues; (2) pelvic inflammatory exudation; (3) the local tissue of the uterus was brittle and dark yellow. This suggests that there are some adverse reactions and complications of ablation therapy that cannot be ignored. Common complications include skin scald, local pain, nerve injury, vaginal fluid, infection, etc. Serious complications include intestinal perforation, uterine bleeding, bladder injury, intestinal obstruction, uterine perforation, etc.^[8,12,15]^ In the study of Zhao,^[12]^ the incidence of minor complications of MWA was 51.70% and that of serious complications was 5%. Li^[15]^ retrospectively studied 1982 cases of adenomyosis after HIFU and found that there was 1 case of lower limb movement disorder, 1 case of urine retention, 2 cases of vaginal bleeding caused by endometrial injury and 1 case of severe complication intestinal perforation. In the study of Qian Yu,^[9]^ abnormal uterine bleeding or pelvic infection occurred in 2 patients after MWA surgery. It is considered that the ablation volume is too large and the endometrium is destroyed, which leads to postoperative ablation area prolongation. For patients who have no requirement for children, the only established treatment option is hysterectomy.^[4]^ In the case, due to the adverse reactions and side effects of ablation therapy, the difficulty of surgical resection is increased and the incidence of complications is increased.

Usually, MWA was performed by ultrasound doctor which lack of follow-up. This case was treated by MWA by the ultrasound doctor in Ultrasound Department, and transferred to the gynecology department to continue treatment. The ultrasound department often has a short follow-up time and lacks comprehensive exploration of pelvic and abdominal conditions during ablation. It is suggested to explore the multidisciplinary team mode of ablation therapy. Multiple combination therapy can compensate for the shortcomings of single ablation therapy through a series of coherent interventions. Studies have shown^[7,9]^ that laparoscopy combined with ablation technology can avoid important organs such as intestinal duct and surrounding large blood vessels during puncture. In addition, the thermal damage of the ablation needle can be avoided by pulling or pressing the surrounding adhesive tissue through exploration forceps. At the same time, the department of ultrasound and obstetrics and gynecology closely cooperate, multi-disciplinary collaboration, more conducive to the realization of complementary advantages.

## 4. Conclusion

This article reports a case of complications after hysterectomy for adenomyosis after MWA, indicating that MWA may not be able to alleviate symptoms in patients with severe adenomyosis, and there is a risk of intestinal adhesions and pelvic infections after MWA surgery. Hysterectomy is more difficult than conventional surgery, requires higher technical skills from the operator, and increases the probability of postoperative complications. Surgical resection should be evaluated comprehensively before proceeding. Especially for patients who have undergone multiple rounds of MWA, multidisciplinary consultations and comprehensive evaluations should be conducted before proceeding with caution.

## Acknowledgments

The authors thank the Department of Network Informatics and the Department of Pathology for providing the relevant medical images. The authors would also like to thank the patient for agreeing to reveal case details for publication.

## Author contributions

**Data curation:** Wenhui Zhao.

**Writing – original draft:** Xiuchun Yang, Shujuan Chen.

**Writing – review & editing:** Jinhong Yang.
